# The comparison between motor imagery and verbal rehearsal on the learning of sequential movements

**DOI:** 10.3389/fnhum.2013.00773

**Published:** 2013-11-18

**Authors:** Arnaud Saimpont, Martin F. Lafleur, Francine Malouin, Carol L. Richards, Julien Doyon, hb Philip L. Jackson

**Affiliations:** ^1^Centre Interdisciplinaire de Recherche en Réadaptation et Intégration Sociale, école de Psychologie, Université LavalQuébec, QC, Canada; ^2^Institut Universitaire en Santé Mentale de Québec, Université LavalQuébec, QC, Canada; ^3^Centre Interdisciplinaire de Recherche en Réadaptation et Intégration Sociale, Département de Réadaptation, Université LavalQuébec, QC, Canada; ^4^Centre de Recherche de l’Institut Universitaire de Gériatrie de Montréal Université de MontréalMontréal, QC, Canada; ^5^Centre de Recherche de l’Institut Universitaire en Santé Mentale de Québec, école de Psychologie, Université LavalQuébec, QC, Canada

**Keywords:** motor imagery, verbal rehearsal, mental practice, foot movement sequence, learning, retention

## Abstract

Mental practice refers to the cognitive rehearsal of a physical activity. It is widely used by athletes to enhance their performance and its efficiency to help train motor function in people with physical disabilities is now recognized. Mental practice is generally based on motor imagery (MI), i.e., the conscious simulation of a movement without its actual execution. It may also be based on verbal rehearsal (VR), i.e., the silent rehearsal of the labels associated with an action. In this study, the effect of MI training or VR on the learning and retention of a foot-sequence task was investigated. Thirty right-footed subjects, aged between 22 and 37 years old (mean: 27.4 ± 4.1 years) and randomly assigned to one of three groups, practiced a serial reaction time task involving a sequence of three dorsiflexions and three plantar flexions with the left foot. One group (*n* = 10) mentally practiced the sequence with MI for 5 weeks, another group (*n* = 10) mentally practiced the sequence with VR of the foot positions for the same duration, and a control group (*n* = 10) did not practice the sequence mentally. The time to perform the practiced sequence as well as an unpracticed sequence was recorded before training, immediately after training and 6 months after training (retention). The main results showed that the speed improvement after training was significantly greater in the MI group compared to the control group and tended to be greater in the VR group compared to the control group. The improvement in performance did not differ in the MI and VR groups. At retention, however, no difference in response times was found among the three groups, indicating that the effect of mental practice did not last over a long period without training. Interestingly, this pattern of results was similar for the practiced and non-practiced sequence. Overall, these results suggest that both MI training and VR help to improve motor performance and that mental practice may induce non-specific effects.

## INTRODUCTION

In the context of motor learning, mental practice may be defined as the cognitive rehearsal of a physical activity in order to enhance performance in this activity (Jackson et al., 2001). Mental practice is generally based on motor imagery (MI), i.e., the mental simulation of an action without its actual execution. Research on mental practice based on MI as a strategy to improve motor performance goes back to the 1930s (e.g., Sackett, 1934) and since then the use of MI training has become widespread in sport settings. It has been shown that it is often better to perform mental practice than no practice and that physical practice combined with mental practice often lead to better results than physical practice alone (see Richardson, 1967a,b; Feltz and Landers, 1983; Driskell et al., 1994; Munzert and Lorey, 2013). Furthermore, there is accumulating evidence that mental practice based on MI can be efficient to help train motor functions in people with physical disabilities of neurological origin (see Dickstein and Deutsch, 2007; Malouin and Richards, 2010; Malouin et al., 2013). The use of mental practice in a rehabilitation setting appears particularly relevant as it provides a unique opportunity to practice different kinds of movements – even complex motor tasks – in an autonomous and safe manner while avoiding undue physical fatigue.

Concerning the underlying mechanisms of MI training, it has been repeatedly shown that MI recruits brain regions pertaining to the motor system (e.g., Decety et al., 1994; Gerardin et al., 2000; Malouin et al., 2003; Munzert and Zentgraf, 2009; Hetu et al., 2013). MI training would thus prepare the body to act by “priming” the brain regions involved in the execution of the action. Furthermore, similar brain plasticity has been demonstrated after physical training and mental training based on MI (Pascual-Leone et al., 1995; Jackson et al., 2003; Debarnot et al., 2011), indicating that both forms of training would involve similar neural mechanisms.

If mental practice generally refers to MI training, it has been proposed that mental practice based on verbal rehearsal (VR) – i.e., the covert repetition of verbal labels attached to different elements of an action – could also be useful to improve motor skills (e.g., Hall et al., 1997). Compared to MI training, however, the use of VR has received considerably less attention. Most of the studies that investigated the use of verbal labels to improve motor skills have explored self-talk strategies by athletes. Self-talk content may be categorized as either motivational or instructional (Theodorakis et al., 2000). Motivational self-talk refers to labels aimed at increasing confidence or motivation (e.g., “you can do it”), whereas instructional self-talk refers to labels aimed at directing attention toward movement cues (e.g., “reach, move right,” etc.) and facilitating the learning of a skill (Zinnser et al., 2006). An important part of the research on self-talk with athletes focuses on its instructional role and it has been shown that the use of verbal labels in this context helps to learn different sport skills (e.g., Ziegler, 1987; Ming and Martin, 1996; Landin and Hebert, 1999). Self talk, however, is most of the time used by athletes in parallel with the movement they actually perform (Gammage et al., 2001), not as a rehearsal strategy used *per se*.

To our knowledge, only one study has investigated the effects of VR on motor learning without simultaneously performing the movements (Hall et al., 1997). Interestingly, this study compared the impact of VR and MI training. Participants were first presented with a series of different patterns of movements, each movement being separated by a period of 15 s. During this 15 s period, depending on group assignment, subjects either (1) imagined the movement twice, (2) repeated a verbal label associated to the movement twice, or (3) imagined the movement once and verbally labeled it once. After the presentation of the movements, subjects performed a puzzle for 10 min, and were then asked to reproduce as many of the movement patterns as they could. The authors found that subjects who had used VR were better than those who had used MI, and that those who employed a combination of the two strategies yielded the best results. Although interesting, the study from Hall and colleagues has several limitations. First, subjects mentally practiced each movement only twice, which is very little in comparison with other studies on mental practice where subjects may mentally rehearse movements for several minutes and across several days (see Schuster et al., 2011). Second, long-term effects of mental practice were not assessed. Finally, the subjects’ performance was essentially assessed by calculating the number of movements recalled as well as the accuracy of the correctly recalled movements, thus by measures of motor memory rather than of motor performance (e.g., speed of execution).

Hence, the main objective of the present study was to compare the effects of mental practice with MI or with VR on the speed to perform a sequential motor task derived from the serial reaction time paradigm (Nissen and Bullemer, 1987). By this way, the impact of VR on motor performance was assessed, and this effect could be directly compared with that of MI training. Another objective was to determine whether these two forms of mental practice led to performance gains that were specific to the practiced sequence. Indeed, it has been shown that mental practice with MI of a finger sequence can be beneficial for both trained and untrained sequences (Nyberg et al., 2006; Olsson et al., 2008). If a non-specific effect was found with both MI and VR, this could indirectly suggest that similar processes are involved during these two forms of mental practice. To achieve these goals, we compared the learning and retention of a sequence of lower-limb movements in three groups of healthy subjects: (1) a group who practiced mentally the sequence using MI, (2) a group who practiced mentally the sequence with VR, and (3) a control group who did not engage in any mental practice condition. The specificity of the effects of practice was tested by measuring subject performance in two conditions that differed with regards to the stimuli used: (1) a practiced sequence and (2) a non-practiced sequence. We hypothesized that, compared to the control condition, mental practice using MI and VR would lead to higher levels of improvement on the task, and that this increase in performance would be more important for the practiced sequence.

## MATERIALS AND METHODS

### SUBJECTS

Thirty healthy right-handed and right-footed subjects ranging in age between 22 and 37 years old (mean = 27.4 ± 4.1 years) were recruited. These participants were assigned to one of three groups: mental practice with MI, MI group (*n* = 10); mental practice with VR, VR group (*n* = 10); and a no mental practice, control group (*n* = 10). All groups were matched with respect to their mean age, years of education, and gender ratio based on analyses of variance and chi-square analyses performed on these variables. The exclusion criteria included major medical problems, neurological disorders, psychological or psychiatric illness, uncorrected hearing impairments, as well as musculoskeletal disorders of the lower limbs. Subjects gave their written consent and were financially compensated for their visits to the laboratory. The study was approved by the ethics committee of the Quebec Institute for Rehabilitation.

### MATERIAL

#### Imaginary distance test

In this test, subjects were asked to imagine walking at a regular pace in a familiar setting using the first-person perspective, and then to judge the distance traveled (see Malouin et al., 2003). They were instructed to imagine walking until the experimenter told them to stop. Unknown to the subjects, each trial was terminated after varying intervals of 15, 25, or 35 s. Administration of these intervals was presented twice and counterbalanced, such that the subjects were not able to predict when to stop walking.

#### Kinesthetic and visual imagery questionnaire

This questionnaire developed by Malouin et al. (2007) assesses MI vividness. It includes 10 items corresponding to 10 basic movements that subjects must execute then imagine in the first-person perspective. In the first part of the questionnaire (visual subscale) subjects try to “mentally see” the movements when they imagine them; in the second part (kinesthetic subscale) they try to “mentally feel” the movements. After each imagined movement, they rate the clarity of the images/intensity of the sensations that they have formed on a 5-point scale, from 1 (no image/no sensation) to 5 (image as clear as seeing/sensation as intense as during physically performing). Note that in the version of the Kinesthetic and Visual Imagery Questionnaire (KVIQ) used in this study the scale was reversed: five corresponded to no image/no sensation and one to the clearest images/most intense sensations. A score for each subscale was calculated (ranging from 10 to 50) then a total score was computed (ranging from 20 to 100).

#### Foot-sequence task

The task was performed in an apparatus that consisted of a pedal (13 cm × 35 cm) mounted in a frame (45 cm long, 29 cm wide, and 60 cm high) that was custom made for this research project. The height and length of the pedal could be adjusted to standardize the foot position relative to the ankle axis of rotation, and the foot was secured by two Velcro straps attached to the pedal. A potentiometer fixed on the pedal axis and connected to an electronic relay box could be adjusted to detect three different pedal angles (positions). The relay box was linked to a computer that generated the auditory stimuli and registered the subject response time (ms) and number of errors.

#### Electromyography

A portable two way electromyography (EMG) device (Pathway MR-20; The Protheus Group) recorded surface EMG activity of two leg muscles, the *tibialis anterior* and the *soleus*, during the different experimental conditions. These EMG recordings served only as a feedback to monitor the absence of muscle contractions during the imagined conditions.

### PROCEDURE

The design comprised a total of seven experimental sessions. Session 1 was a pre-training and baseline evaluation, sessions 2–6 were weekly evaluations of subjects’ physical and mental performance, while session 7 was conducted several months after session 6 to assess the retention level of the skill. Between sessions 1 through six, subjects in the MI and VR groups mentally practiced a specific sequence of six elements. Subjects in the control group did not practice between sessions, but their performance was nevertheless tested at the same time points as the other two groups.

#### Testing session 1

After the procedure was fully described to the subjects, participants completed the imaginary distance test as a preparation for MI. Due to time constraints the KVIQ was handed to them to be completed at home and returned in the next session.

***Execution of the foot-sequence task***.Participants were set up in the apparatus with their left foot attached to the pedal, and were asked to perform the task in a supine position. Note that the left foot was used because performance with this limb was expected to offer more room for improvement than with the right limb. Three foot positions were determined: (1) maximum dorsiflexion (up), (2) middle position, and (3) maximum plantar flexion (down). The relay box was adjusted to recognize these positions. Subjects started the task with their foot in the middle position. They were requested to execute a dorsiflexion in response to a high pitched sound and a plantar flexion in response to a low pitched sound. They were required to move as quickly as possible while making as few errors as possible. After each trial, subjects had to move their foot back to the middle position in order to be ready to move in response to the upcoming target sound. The trials were presented with a fixed inter-stimulus interval of 2000 ms.

Each subject was given 24 practice trials to become familiar with the physical execution of the task. They were then asked to complete trials in two different conditions: sequence A and sequence B. The order of presentation of the two sequences was counterbalanced among groups. In addition, random trials were administered between the two conditions to reduce possible confusion between the two sequences (results for these trials were not included in the analyses). Sequence A corresponded to the following sequence of six foot positions: “up-down-down-up-down-up,” while Sequence B consisted of the reverse order: “down-up-up-down-up-down.” These sequences were found to be equivalently difficult in a previous pilot experiment (data not shown here). Four blocks were performed with one of the sequences, followed by two blocks of the random trials, and then four blocks of the alternate sequence. Each block consisted of 36 trials (6 sequences of 6 elements), and were separated by 1 min pauses. Before training began, subjects were taught explicitly the series of movements and had to reproduce it* errorless *three times in a row without any auditory cues. The response time (ms) was recorded for each trial.

***Imagination of the foot-sequence task.*** Following assessment of the initial performance on the foot-sequence task, two electrodes were attached to the subject’s left leg over the *tibialis anterior *and the *soleus* muscles to record EMG activity during the covert conditions. If such activity was present, subjects were asked to relax, and repeat the imagined block of trials until no significant change in the EMG signals was observed. During testing, subjects in the MI and control groups had to imagine the movements of the sequence, while those in the VR group had to mentally repeat the labels “up” and “down” associated with the sequence. Precisely, during MI, subjects were asked to imagine, as quickly and accurately as possible, four blocks of six sequences for both Sequence A and B, starting with the one they began with during physical execution of the task. MI involved the kinesthetic and visual components of the movements as if subjects were performing the task (first-person perspective). For its part, VR consisted of a silent repetition of the sequence of foot positions (i.e., “up-down-down-up-down-up” and “down-up-up-down-up-down”). After the start signal, subjects with their eyes closed, counted on their fingers the number of sequences they performed mentally, to indicate the exact moment they completed one block (six sequences) of trials. The time, in seconds, taken to imagine each block was recorded with a stopwatch.

#### Mental practice

Subjects in the MI and VR group were asked to complete 12 practice periods at home before coming to the next testing session. During each practice period, they had to assume a sitting or supine position, and imagine/labeling the sequence without actually moving their foot for 10 separate blocks of trials (10 × 6 sequences = 60 sequences per practice period). Thus, subjects in the MI and VR groups mentally rehearsed 720 times their practiced sequence between each testing session. Subjects were given a logbook in which they were asked to register the time and duration of each training period. Subjects in the control group were not asked to practice the sequences but returned to the laboratory for weekly evaluations.

#### Testing sessions 2–6

During each testing session, which took place on average 8.4 days (SD = 1.7) apart, subjects had to perform the foot-sequence task both physically and mentally as described previously in the first testing session, except that only two blocks of practice of each sequence (instead of four) were administered. Therefore, all subjects were tested on the foot-sequence task on two blocks of the practiced sequence and two blocks of the non-practiced sequence, separated by two blocks of random trials. Again, subjects in the MI and control groups were asked to perform the task using MI, while subjects in the VR group were required to use covert VR. EMG activity of the *tibialis anterior *and *soleus* muscles was recorded again in session 6 to insure that repeated mental practice did not induce muscular activity. At the end of session 6, subjects were again given the KVIQ to be completed at home in the next few days to determine whether the perception of their imagery ability had changed after several weeks of mental practice.

#### Testing session 7

All of the subjects who participated in this experiment were later invited to come back to the laboratory for a retention test. Subjects were not previously told about this test to insure that no further practice would occur after training sessions. Twenty-three subjects (MI group, *n* = 9; VR group, *n* = 8; control group, *n* = 6) were re-tested on average 206 (SD = 46) days after session 6. They completed two blocks of the practiced sequence and two blocks of the non-practiced sequence, separated by two blocks of random trials. They also imagined two blocks of each sequence as described previously.

### DATA ANALYSIS

#### Motor imagery ability

The total KVIQ-scores obtained during the first administration of the questionnaire were compared between groups by means of a one-way ANOVA. Further, to determine whether mental practice of a skill during several weeks altered the perception of imagery ability, we compared KVIQ scores at the beginning of the experiment with those obtained after practice by means of a 2 × 3 (Session × Group) ANOVA with repeated measure.

#### Execution of the foot-sequence task

Only response times were analyzed since subjects made very few errors (mean: 1.6%). First, response times shorter than 100 ms and longer than 2000 ms were discarded. Indeed, it has been shown that genuine reaction times cannot be less than 100 ms (e.g., Luce, 1986) and the cut-off value of 2000 ms was chosen to eliminate trials were subjects erred. Then, response times inferior or superior to two SD of the subject’s mean for a given condition were excluded. On this basis, results for one subject from the control group were not included in the analyses because almost 25% of trials were outliers, which strongly suggests that this participant did not fully comply with the instructions. For the other subjects, no more than 8% of the trials were discarded (mean: 2.4%). Response times were compared between groups, for each condition, at baseline (session 1) after 5 weeks of training (session 6), and around 6 months after the end of training (session 7, retention) by means of ANOVAs (See Results).

#### Imagination of the foot-sequence task

To explore the temporal congruence between executed and imagined movements, we compared the time taken to physically and mentally complete the blocks of sequences. A first descriptive analysis of the data led to an unexpected finding. In fact, individual data showed that some subjects tended to take more time during imagination than during execution of the sequence (over-estimators), some subjects took approximately the same amount of time, while others took less time to imagine the task than to execute it (under-estimators). Moreover, subject tendency to use a given strategy during MI was found to be relatively constant across training. Since the distribution of this unsuspected characteristic was not balanced across groups (e.g., four over-estimators in the MI group, and seven in the control group), comparison between the time taken by subjects to imagine and execute the foot-sequence task was not pursued.

## RESULTS

### MOTOR IMAGERY ABILITY

Results from the imaginary distance test confirmed that all subjects understood the concept of MI and were able to imagine movements. Indeed, consistent with previous studies (Malouin et al., 2003, 2008), subjects imagined walking farther with increasing time and vice-versa. Also, all subjects succeeded in engaging in MI of the foot-sequence task without significantly contracting their *tibialis anterior* or *soleus* muscles either during the first or sixth session.

As for the KVIQ, the one-way ANOVA performed on the scores obtained during the first administration of the questionnaire showed a significant main effect of Group [*F*_(2,26)_ = 6.13, *p* < 0.01]. Decomposition of this effect revealed that the control group scored significantly lower – thus rated itself as being better at eliciting vivid images and sensations – than both the MI and VR groups (*p* < 0.05). To determine whether mental practice of a skill during several weeks altered the subjects’ perception of their imagery ability we compared the KVIQ scores between sessions 1 and 6 by means of a 2 × 3 (Session × Group) ANOVA with repeated measures. The main effect of Session, as well as the Group × Session interaction failed to reach the level of significance, suggesting that subjects, on average, did not change their rating of their own MI ability over time.

### EXECUTION OF THE FOOT-SEQUENCE TASK

**FIGURE 1:**
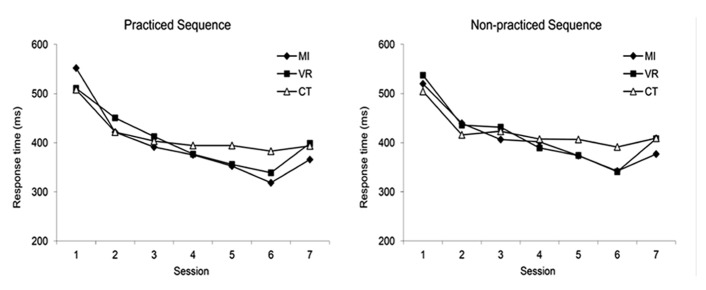
**Mean response times for the practiced and non-practiced sequence, in each group, across the seven sessions**.

Six subjects (one in the MI group, two in the VR group, and three in the control group) did not attend the retention session for different reasons (could not be contacted, refused to come back). **Figure 1** provides an overview of the evolution of the mean response times for the practiced and non-practiced sequence, in each group, across the seven sessions. Response times for both conditions decreased in all groups between sessions 1 and 6, and this decrease was more important in the MI and VR groups compared to the control group. Also, while response times remained relatively stable between sessions 6 and 7 in the control group, they increased in the MI and VR groups. In the following sections, we provide statistical analyses of these data. The performance change between sessions 1 and 6 were analyzed separately (*n* = 29) from the performance change between sessions 6 and 7 (*n* = 23).

#### Training: performance change between sessions 1 and 6

**FIGURE 2:**
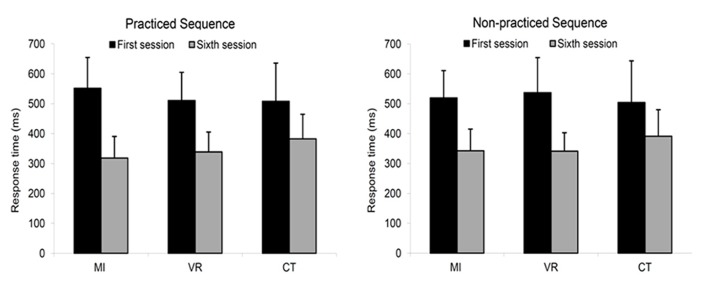
**Mean (+SD) responses times for the practiced and non-practiced sequence, in each group, at sessions 1 and 6.** MI, Motor imagery group; VR, verbal rehearsal group; CT, control group.

First, a one-way ANOVA conducted on responses times at session 1 showed that the performance levels before training did not differ significantly between groups [*F*_(2,26)_ = 0.51, *p* = 0.608]. Thus, possible differences between groups after practice should reflect the effects of the different training regimen. **Figure 2** shows mean responses times for the practiced and non-practiced sequence, in each group, at sessions 1 and 6. Results of a 3 × 2 × 2 (Group × Condition × Session) ANOVA performed on the response times showed a significant main effect of Session [*F*_(1,26)_ = 150.51, *p* < 0.001, $\eta_{p}^{2}$ = 0.85] as well as a significant Group × Session interaction [*F*_(2,26)_ = 3.40, *p* < 0.05, $\eta_{p}^{2}$ = 0.21], indicating that the three groups improved their performance after training, but that this change in performance differed among groups. Subsequent paired *t*-tests with a Sidak correction conducted within each group showed that response times for both sequences significantly decreased between session 1 and 6 in the three groups (*p* < 0.001). To further characterize the Group × Session interaction, we thus conducted three separate 2 × 2 (Group × Session) ANOVAs on data of the groups taken two by two (MI vs. control, VR vs. control, and MI vs. VR). Results showed that the interaction between Group and Session was significant when the MI group was compared to the control group [*F*_(1,17)_ = 7.00, *p* < 0.05, $\eta_{p}^{2}$ = 0.29], approached significance between the VR and control groups [*F*_(1,17)_ = 7.00, *p* = 0.067, $\eta_{p}^{2}$ = 0.18], but was not significant between the MI and VR groups. Note that in the 3 × 2 × 2 (Group × Condition × Session) ANOVA, neither the effect of Condition, nor any other interaction involving the effect of Condition reached statistical significance, suggesting that the changes in response times were not statistically different between the practiced and non-practiced sequence.

#### Retention: performance change between sessions 6 and 7

**FIGURE 3:**
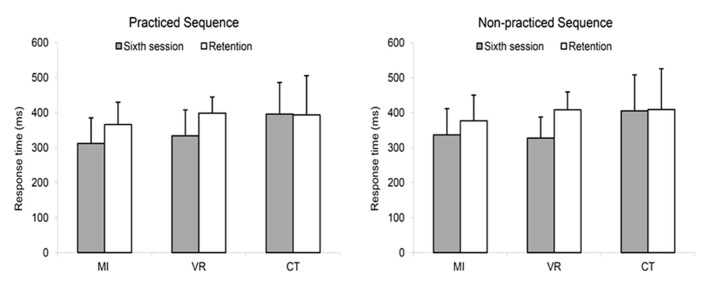
**Mean (+SD) responses times for the practiced and non-practiced sequence, in each group, at sessions 6 and 7 (Retention).** MI, Motor imagery group; VR, verbal rehearsal group; CT, control group.

**Figure 3** shows mean responses times for the practiced and non-practiced sequence, in each group, at sessions 6 and 7. The results of a 3 × 2 × 2 (Group × Condition × Session) ANOVA performed on the response times showed a significant main effect of Session [*F*_(1,20)_ = 32.42, *p* < 0.001, $\eta_{p}^{2}$ = 0.62] as well as a significant Group × Session interaction [*F*_(2,20)_ = 8.06, *p* < 0.01, $\eta_{p}^{2}$ = 0.45], indicating that there was a change in performance after several months without practice but that this change differed between groups. In fact, paired *t*-tests with a Sidak correction conducted within each group revealed that response times for both sequences significantly increased (*p* < 0.001) in the MI and VR groups but did not change in the control group (but remember that the control group had not improved as much as the other groups in the training phase). This result showed that the additional gain in performance obtained after training in the two mental practice groups compared to the control group did not last after several months without practice. To further investigate whether the change in response time differed between the MI and VR groups, we performed a 2 × 2 (Group × Session) ANOVA on the data of these two groups. This analysis did not show any significant interaction between Group and Session, indicating that the decrease in performance between sessions 6 and 7 was equivalent for the MI and VR groups. Finally, note that in the 3 × 2 × 2 (Group × Condition × Session) ANOVA, no interaction involving the effect of Condition reached statistical significance, showing that the changes in response times were equivalent between the practiced and non-practiced sequence.

## DISCUSSION

The main results of this study showed that, compared with no mental training, both mental practice with MI and with VR enhanced performance of a sequential motor skill after a few weeks of training. Furthermore, the two conditions of mental practice led to improved performance of the trained sequence as well as an untrained sequence, suggesting a non specific effect of training. After several months without mental practice however, performance returned to similar levels in the mental training groups and the control group, indicating that the gain provided by mental practice after 5 weeks of training was not maintained over time.

### EFFECTS OF MENTAL PRACTICE ON MOTOR LEARNING

#### Mental practice with motor imagery

When considering the effects of mental practice with MI, our findings are consistent with a large body of research in sport settings, which support the use of MI training to improve the learning of sequential skills (see Feltz and Landers, 1983; Driskell et al., 1994). As shown by other studies, it is also possible to improve motor sequence skills with mental practice based on MI even without the extrinsic motivation of competitive athletic performance (Pascual-Leone et al., 1995; Jackson et al., 2003; Gentili et al., 2006; Nyberg et al., 2006; Olsson et al., 2008; Debarnot et al., 2009; Debarnot et al., 2011). For example, by using the same task as the one of the present study, Jackson et al. (2003) showed that five training periods of mental practice with MI over 1 week led to a significant improvement in performance in healthy young subjects. For their parts, Olsson et al. (2008) showed that a combination of physical and mental practice of a finger tapping sequence over 6 weeks tended to induce greater improvement in the speed of execution of the sequence than physical practice alone. In the present study, subjects in the MI group combined physical and mental practice since they executed the sequence at each testing session. Our results are thus directly in line with those of Olsson and colleagues and extend them to the learning of a sequence involving the lower limb.

Such improvements in performance after mental practice have been linked with changes in the cortical maps associated with the movements performed (e.g., Pascual-Leone et al., 1995; Jackson et al., 2003; Debarnot et al., 2011). For example, in the study by Jackson et al. (2003) mentioned above, the authors showed that the performance improvement after mental practice was accompanied by an increase in activity in the orbitofrontal cortex as well as a decrease in activity in the cerebellum, both changes in the functional representation of the skill that had previously been shown to occur after physical practice of the same task (Lafleur et al., 2002). Hence, mental practice with MI of sequential skills can access and modify the motor representation of the practiced skills, just like physical practice.

#### Mental practice with verbal rehearsal

It is of interest that our results also show improvements in performance after VR that were similar to those obtained with MI training. VR as a technique to improve motor skills has been considerably less studied than MI training, even in athlete populations. Furthermore, although the impact of VR training has generally been shown to be positive, it was essentially in a context where subjects labeled key elements (via self-talk) of a given movement concomitantly to its actual execution (e.g., Ziegler, 1987; Ming and Martin, 1996; Landin and Hebert, 1999; Zinnser et al., 2006). To our knowledge, the only published study that specifically explored the impact of VR as a training technique decoupled from actual execution was that of Hall et al. (1997) where the effects of mental practice with VR and MI on the memorization of different movements were compared. However, as reported in the introduction, the study of Hall and colleagues had important limitations and notably the fact that subjects mentally practiced the movements only twice and that the main outcome was a measure of recall. Our results thus add an original contribution to the literature on mental training as they show that the increase in speed – a real measure of motor performance – in a sequential motor task was similar after a substantial amount of VR and after the same amount of MI training. Note however that the present results do not allow us to conclude that VR (combined with physical practice) is truly more efficient than physical practice alone since the difference in the performance improvement between the VR and control groups only approached significance (whereas this difference was significant between the MI and control groups). The impact of VR on motor performance needs thus to be further investigated.

Still, one possible explanation of the effect of VR comes from the links between language and movements, as for example proposed in the action-language-imagination model by Annett (1996). According to this model, there are two main channels to acquire information about a skill: a motor channel and a verbal channel. Between the two channels is the action-language bridge which makes it possible to verbally describe an action but also to generate an action on verbal instructions. Assuming the existence of such a close relationship between language and movements, it is thus conceivable that by rehearsing the different foot positions, subjects implicitly evoked part of the action itself, thereby engaging motor representations involved in motor sequence learning. More recently, embodied theories of language have put forward the notion that brain areas involved in perception and action are also implicated in the representation and processing of language (e.g., Pulvermuller and Fadiga, 2010). In particular, neuroimaging studies have shown that the processing of action-related language – such as action words – recruits sensorimotor brain areas similar to those that would be activated during actual execution of the actions described by the words (e.g., van Dam et al., 2010; Hauk and Pulvermuller, 2011).

In the present study the words “up” and “down,” although they were not action verbs, clearly referred to an action. It is thus possible that motor representations of the movements were implicitly evoked when subjects mentally rehearsed these words. However, this remains speculative. In future studies, it would be interesting to directly test whether MI of a given action and the processing of key words associated with this action would activate similar brain regions. Furthermore, brain plasticity associated with both VR and MI training should be explored.

### NON-SPECIFIC LEARNING EFFECT

An interesting finding of this study is that the level of improvement was similar for the practiced and the non-practiced sequence, after both MI and VR training. Nyberg et al. (2006) assessed performance of two finger-tapping sequences before and after 1 week of training in two groups of subjects. During training, all subjects practiced one of the two sequences for four sessions spread over 1 week. Half of the participants performed physical practice while the other half performed mental practice based on MI. A positive effect of training (either physical or mental) was shown for both the trained and untrained sequence, although the gain in performance was significantly larger for the trained sequence. In a subsequent study, by using the same finger-thumb opposition task, Olsson et al. (2008) showed that a combination of physical and mental practice for 6 weeks induced a significant increase in tapping performance for the trained sequence, but also, to a lesser extent, for an untrained sequence. Thus, the present results are in line with these findings and extend them, since they show that the trained and an untrained sequence may equally benefit from mental practice.

It has been shown that the learning of the abstract structure of a sequence (i.e., the relationship between repeating elements) can be generalized to an isomorphic sequence (Dominey et al., 1998). For example, the sequences ABCABC and DEFDEF share the same format as they follow the rule 123123 (Dominey et al., 1998). Considering that the two six-element sequences used in the present experiment shared the same abstract structure (i.e., 122121), part of the non-specific learning observed might be due to the acquisition of that structure, an acquisition that could have helped the anticipation of the subsequent element of the sequence even in the untrained sequence. Also, as the learning of a sequence develops, its coding in motor coordinates develops (Hikosaka et al., 2002), thus reducing the speed to perform each movement of the sequence. The “strengthening” of the specific motor representation for the dorsiflexion and the plantar flexion during mental practice of the specific sequence could thus have been transferred to the non-specific sequence since both sequences were composed of the same two movements.

Finally, it is also very interesting that mental practice based on MI and on VR induced a similar non-specific effect. This latter result suggests that similar processes are potentially engaged during MI training and VR.

### RETENTION AFTER SEVERAL MONTHS WITHOUT PRACTICE

Another novel finding of this study is that there was a similar decrease in performance in the MI and VR groups after about 6 months without practice, whereas no significant decrease was observed in the control group (who had improved less than the other groups during training). Hence, the level of performance in the three groups was equivalent at the retention session. This suggests that physical practice was the key element for long-term retention of this sequential motor task, and that MI and VR offered a boost in performance that was present during training only. To our knowledge, this is the first time that mental practice effects were assessed at such a long-term follow up. Most of the studies on mental practice with MI, either in Sport, Psychology, or Medicine, have investigated learning effects of MI training, with simple pre-training/post-training designs (see Schuster et al., 2011). Hence, this result raises a potentially important limit of mental practice, at least for a sequential motor task that must be performed at maximum speed. Further studies should explore long-term effects of mental practice on different parameters of motor performance, such as accuracy or strength. Finally the fact that the decrease in performance at retention was similar in the MI and VR groups once again suggests that similar processes could be involved in both forms of mental practice.

### MOTOR IMAGERY

Beyond the investigation of the impact of mental practice, our results also add to the literature on psychophysical studies of MI. Several chronometric studies have shown that the time taken to imagine a movement is similar to that taken to execute the same movement (Decety et al., 1989; Sirigu et al., 1995; Papaxanthis et al., 2002; see Guillot and Collet, 2005). However, while this might be true at the level of group analysis, comparison of individual data from the actual and imagined sequences in the present study showed that each subject had his or her own strategy for imagining movements, and that this strategy remained fairly constant from one session to the next. Indeed, we found that, on an individual basis, subjects often under-estimated or over-estimated the time it took to actually complete the sequences during MI of the task. In addition, subjects remained either under-estimators or over-estimators even after extensive MI training. This suggests that the temporal congruence between imagined and executed performance is related to individual differences and that it does not only reflect the level of MI ability *per se*. Finally, the lack of significant changes in the KVIQ scores after extensive MI training also supports the notion that some characteristics of subjects’ MI ability (in this case their subjective rating of MI vividness) are relatively stable, at least over a few weeks.

### LIMITS

At least two limitations of this study should be acknowledged. First, the number of subjects included was relatively small. In particular, the fact that seven subjects did not attend the retention session leads us to interpret the retention results with some caution. Second, mental practice was performed at home and although subjects reported to comply with the instructions not to move during practice, EMG activity was not controlled during the training sessions. It is thus possible that some subjects may have not totally inhibited their movements during MI training. Note however, that subjects imagined the sequence without any EMG activity at sessions 1 and 6.

## CONCLUSION

Taken together, the results of the present experiment show that mental practice based on MI and on VR improved the speed to perform a sequential motor task and that this improvement was similar between the MI and VR groups. Although further research is needed to confirm the impact of VR on motor performance, the present results thus suggest that VR could be a useful alternative to MI when using mental practice. It is now well established in the literature that the use of mental practice with MI can provide an adjunct to traditional physical therapy in a rehabilitation setting where specific series of movements often need to be learned or re-learned (see Dickstein and Deutsch, 2007; Malouin and Richards, 2010; Malouin et al., 2013). However, some neurological patients could encounter more difficulties to imagine movements (which is a cognitively demanding activity) than labeling them. The use of VR instead of MI during mental practice – at least in the first stages of mental training – with these patients could thus be of particular interest; this remains to be tested.

## Conflict of Interest Statement

The authors declare that the research was conducted in the absence of any commercial or financial relationships that could be construed as a potential conflict of interest.
